# Photonic neuromorphic technologies in optical communications

**DOI:** 10.1515/nanoph-2021-0578

**Published:** 2022-01-19

**Authors:** Apostolos Argyris

**Affiliations:** Instituto de Física Interdisciplinar y Sistemas Complejos IFISC (CSIC-UIB), Campus UIB, Palma de Mallorca, 07122, Spain

**Keywords:** fiber transmission, machine learning, neuromorphic computing, optical communications, photonic systems, reservoir computing

## Abstract

Machine learning (ML) and neuromorphic computing have been enforcing problem-solving in many applications. Such approaches found fertile ground in optical communications, a technological field that is very demanding in terms of computational speed and complexity. The latest breakthroughs are strongly supported by advanced signal processing, implemented in the digital domain. Algorithms of different levels of complexity aim at improving data recovery, expanding the reach of transmission, validating the integrity of the optical network operation, and monitoring data transfer faults. Lately, the concept of reservoir computing (RC) inspired hardware implementations in photonics that may offer revolutionary solutions in this field. In a brief introduction, I discuss some of the established digital signal processing (DSP) techniques and some new approaches based on ML and neural network (NN) architectures. In the main part, I review the latest neuromorphic computing proposals that specifically apply to photonic hardware and give new perspectives on addressing signal processing in optical communications. I discuss the fundamental topologies in photonic feed-forward and recurrent network implementations. Finally, I review the photonic topologies that were initially tested for channel equalization benchmark tasks, and then in fiber transmission systems, for optical header recognition, data recovery, and modulation format identification.

## 1 Introduction

Over the last decades, there have been major technological breakthroughs in fiber-optic systems, which have shaped the current capabilities of the data transfer network infrastructure. Since the first designs and fabrication of optical fibers [1], several technological stepping stones consolidated the fiber-optic technology and network operation, as we know it today. The development of erbium-doped fiber amplifiers (EDFA) [2] offered the elimination of electrical-optical conversions. The enabling of wavelength-division multiplexing (WDM) [3] allowed to exploit the same physical medium under extended parallelism. The development of trustworthy coherent receivers [4] allowed us to increase the dimensionality of the encoded information and the transmission reach. And finally, DSP led to an explosion of the overall data throughput over the physical fiber channel [5]. The latest research pathways in optical communications introduce now new challenges; encoding information with high-order modulation formats, transmitting signals with optical power beyond the fiber nonlinearity limit, improving the signal-to-noise ratio (SNR) at the receiver, and establishing phase noise and inter-channel interference compensation for high-baud rate systems [6]. In parallel, novel designs of physical components respond to these challenges, while reducing their energy and size footprint. New optical sources aim at low relative intensity or/and phase noise generate optical carriers. Optical modulators are optimized to support multi-level modulation while optimizing the resulting frequency-chirp of the modulating signal and operating at low bias voltage. Optical amplifiers are improved in terms of their noise figure. At the detection stage, photoreceivers with high bandwidth, flat response, and high responsivity are re-designed.

But apart from the advances that apply to the physical channel, major research efforts focus on the data encoding and decoding methodologies. The regulation of the propagating signal properties has proven beneficial to annul or partially mitigate transmission-induced effects. At the emitter, pulse shaping is used to tune the form of the physical signal that represents the encoded binary digits or symbols. Especially for multi-level, amplitude-phase (IQ) data encoding schemes, this was commonly addressed by probabilistic shaping (PS) or geometric shaping (GS) [5, 7], [8], [9], aiming to approach the Shannon limit [10]. The most appropriate shaping is defined by iterative optimization algorithms, which aim at minimizing the average symbol error probability and maximizing the mutual information [11]. At the receiver, multiple post-processing stages were used over the years to improve the quality of data recovery. For systems without physical compensation of the chromatic dispersion, digital filter equalizers were implemented [12], [13]. But most challenges arise when aiming at the compensation or mitigation of the fiber nonlinear effects. Intra- and inter-channel nonlinearities in the optical fiber are the main impairments that limit the reach of a transmission line [5, 14, 15]. The established algorithmic solutions nowadays include perturbation solutions to the coupled nonlinear Schrödinger equation (CNLSE), single-channel and multi-channel digital backpropagation (DBP) [16], Volterra nonlinear equalizers [17], and Kramers–Kronig receivers [18]. Independently of the approach, the computational complexity of the DSP algorithms needs to be preserved at a level that allows the receiver to operate at the rate of the optical communication system. But since the data encoding rates in modern communication systems go along with the technological limitations, the real-time implementation of the above algorithms is arduous. In tasks that make use of the statistical properties of the transmitted signals – such as optical network monitoring, validation of the integrity of the network operation, and detection of faults in data transfer [19], [20], [21], [22], [23] – the use of complex and time-expensive algorithms is not a problem. Operating tests that are diagnostic and performed occasionally allow for computationally heavy post-processing. Thus, a plethora of complex algorithms have been employed to this end, including ML techniques and deep neural networks [24], [25], [26], [27].

Over the last decades, significant efforts were made in the direction of exploiting the optical components, which were already used in optical communication systems, at various stages of signal processing. For example, fiber-based topologies were used for optical signal regeneration, by using the nonlinear four-wave mixing effect [28], and fiber Bragg gratings were used as power equalizers and chromatic dispersion compensators [29]. However, photonic systems can offer more sophisticated and efficient computing possibilities. The idea of optical computing is not at all new [30]. But it was only until the last decade when an exploding number of various photonic implementations were proposed, based on fiber optics [31], free-space optics [32], multimode fibers [33], and photonic integrated circuits [34]. Several photonic and optoelectronic systems with minimalistic hardware designs were studied, demonstrating their capability to trigger complex behaviors. Time-delayed nonlinear optical [35] and optoelectronic [36] oscillators were extensively explored, both theoretically and experimentally, providing significant insights into their dynamical properties. Besides their dynamical investigation, these systems were the core of a wide range of applications, including optical chaos communications, physical random number generation, broadband microwave signal generation, and sensing [37], [38], [39], [40], [41]. Such dynamical systems were the basis for the first analog neuromorphic optical computing studies, and, specifically, the demonstration of reservoir computing (RC) [42, 43] by engaging photonic devices [31], [44], [45]. Their key functionality to perform computing tasks was the nonlinear transformation of the input information and the supervised or unsupervised learning of the output information. These systems were tested in applications such as handwritten digit recognition, speech recognition, image classification, etc., with quite relaxed speed requirements in processing. However, a speed bottleneck in signal processing may occur at different stages of an optical computing system. One example is the use of spatial light modulators at the input interface for information encoding, in spatial or spatiotemporal optical computing systems, limiting the processing rate to the kHz range. Another example is the use of photonic and optoelectronic devices with limited bandwidth for nonlinear transformations, at the photonic processing stage. Consequently, converting the optical computing concepts into processing tools for ultrafast information processing is a challenge. In optical communications, the main target of such systems would be to perform on-the-fly signal processing with increased complexity and not suffer from a computational speed penalty.

In the next section, I provide an extended introduction of the latest DSP approaches that incorporate ML algorithms and are tested in various optical transmission systems. Some of these approaches, of low complexity, are being used in actual implementations. In Section 3, which is the main part of this review, I present the neuromorphic analog photonic approaches that were recently proposed as an alternative to DSP algorithms, with the aim at transferring some of the signal processing to the analog domain.

## 2 Machine learning in optical communications

The mitigation of linear and nonlinear effects in fiber transmission systems is a field where the signal processing community put huge research efforts in the last decades. Some of them made use of the nonlinear Schrödinger equation (NLSE) [46], which emulates the propagation along the fiber-optic channel. Other models exploited DBP, a method that followed the inverse directionality in signal propagation, from the receiver to the transmitter, to compensate deterministic effects [16, 47, 48]. However, the contribution of stochastic processes usually was not taken into account, while the algorithmic complexity of these models did not offer practical solutions [14]. Simpler approximations of the NLSE were considered, such as Kalman [49] and Volterra [50] filters, which were closer to the realistic applications and appear now as the conventional DSP approaches. The nonlinear Fourier transform (NFT) [51], [52], [53], [54] has also received significant attention as a computational tool against the induced nonlinearities of fiber transmission [55]. But in an overall assessment, the DSP approaches are either of very low complexity and with limited performance, or they engage complex algorithms that describe better the transmission channel and perform more efficiently. In the first case, they can apply in real-time systems, but in the second case, they are too complex and computationally expensive. To this end, the expectations for ML to handle more complex problems with simpler implementations schemes were reasonably high. During the last decade, numerous works employed ML for the characterization and operation of individual components, the quality of transmission, and the network monitoring, but also the mitigation of linear and nonlinear effects. The large number of recent articles, that reviewed extensively the research of the above topics, shows the impact of ML on the field of optical communications [14, 20, 24, 25, 27, 56, 57].

For the mitigation of channel impairments, the nonlinear transmission effects were addressed in both direct-detection and coherent-detection systems. In direct-detection systems, NN-based equalizers were studied, based on multi-layer perceptrons [58], convolutional NNs (CNN) [59], echo state networks (ESN) [60], and long short-term memory NNs [61]. For example, Karanov et al. [62] demonstrated an artificial NN (ANN) transceiver for nonlinear compensation in a 42 Gb/s, 40 km intensity-modulation/direct detection (IM/DD) system. In [63], Ranzini et al. compared a NN equalizer with hyperbolic tangent activation functions, showing that the transmission reach can be almost doubled. They also showed that RC-based and FNN equalizers as DSP blocks exhibit similar performance. In coherent systems, approaches based on black-box [64], channel-based [65, 66], or analytical models were investigated, targeting also long-haul transmission. For example, Zibar et al. [67] used an expectation-maximization algorithm to learn the channel properties from the demodulated data. Li et al. [68] introduced SVM supervised learning to mitigate the nonlinear phase noise in a signal-carrier 16-QAM coherent optical system. Giakoumidis et al. used an SVM [69] and an ANN [70] nonlinear equalizer with a classifier of reduced complexity, in a 16-QAM 2000 km transmission experiment, outperforming a Volterra nonlinear equalizer, exhibiting low computational load and execution time. In adaptive versions of the DBP, Oliari et al. [71] used a learned DBP approach, where the linear steps in the split-step method were re-interpreted as general linear functions, similar to the weight matrices in a deep neural network. In [72], Fan et al. optimized the standard DBP as a deep neural network (DNN) in an 815 km long-haul transmission system. In [73], Sidelnikov et al. used a CNN-based equalizer for compensating nonlinear signal distortions in a 3200 km 16QAM long-haul transmission link, with 4 times less complexity than a multi-channel DBP-based scheme.

Besides the application of ML tools for channel equalization and mitigation of nonlinear transmission effects, several studies lately addressed the modulation format identification, as a computing task. In most of the research studies in fiber transmission systems, the modulation format of the encoded information is predetermined. However, in real operating networks, there is not always an *a priori* knowledge of the modulation encoding format of the processed signal. The sequential data packets that arrive at a routing element may transfer information that corresponds to different protocols and encoding formats. This makes the management of such heterogeneous optical networks a challenging and complex task. In practice, simple techniques which are based on features extraction are currently used. For example, the normalized power distribution [74] or the entropy of the amplitude histogram [75] of the received signals may be used to distinguish the different formats. Some more sophisticated proposals incorporated ML architectures [76]. In some examples, Borkowski et al. [77] presented a Stokes-based algorithm in a digital coherent receiver, which did not require any training or a reference constellation diagram to operate. Khan et al. [78] proposed the use of an ANN, trained with the features extracted from the asynchronous amplitude histograms of the directly detected signal. In [79], Khan et al. applied DNNs for pattern recognition on the histograms of the previous work, which were obtained after constant modulus algorithm equalization. In [80], Tan et al. used principal component analysis (PCA) to identify the modulation format and the data rate of the encoding. Wang et al. employed in [81] a CNN for feature extraction and self-learning from the raw image of the corresponding eye diagram.

All these works demonstrate the algorithmic computational capability of ML to contribute and improve the current state of the art of DSP in optical communications. In principle, all the above implementations, as well as many others that were announced in the last years, target on efficient signal processing while requiring less computational complexity compared to the conventional DSP methods. Still, many of the proposed ML solutions are too complex to be implemented in real-time. In fact, several of them perform better but are even more complex when compared to standard DSP solutions. Moreover, many of the proposed schemes require high computational power. Thus, several ML concepts that are too demanding for algorithmic implementation have been lately considered in analog photonic implementations.

## 3 Photonic neuromorphic computing in optical communications

It was only the last decade when the first designs and implementations in photonic or optoelectronic reservoir computing were proposed. And while most of them evaluated their classification or prediction performance in various static benchmark tasks, one of them was related to signal transmission, and specifically to channel equalization. Lately, these configurations were tested in some challenging tasks in optical communication systems. These include the optical header recognition, the modulation format identification, and the data recovery from transmission systems in presence of linear and nonlinear effects. In this section, I review the different photonic topologies that tackle the aforementioned challenges and are proposed as an alternative to the software-based DSP algorithms. I focus on the neuromorphic implementations that are based on photonic RC since this computing simplification was the basis of almost all relevant works in the field. The considered RC topologies utilize either multiple nonlinear elements with recurrent connections, emulating the conventional neural nodes, or a single nonlinear photonic element with time-delayed connectivity [44, 45, 82].

### 3.1 Connectivity topologies

**Figure 1:**
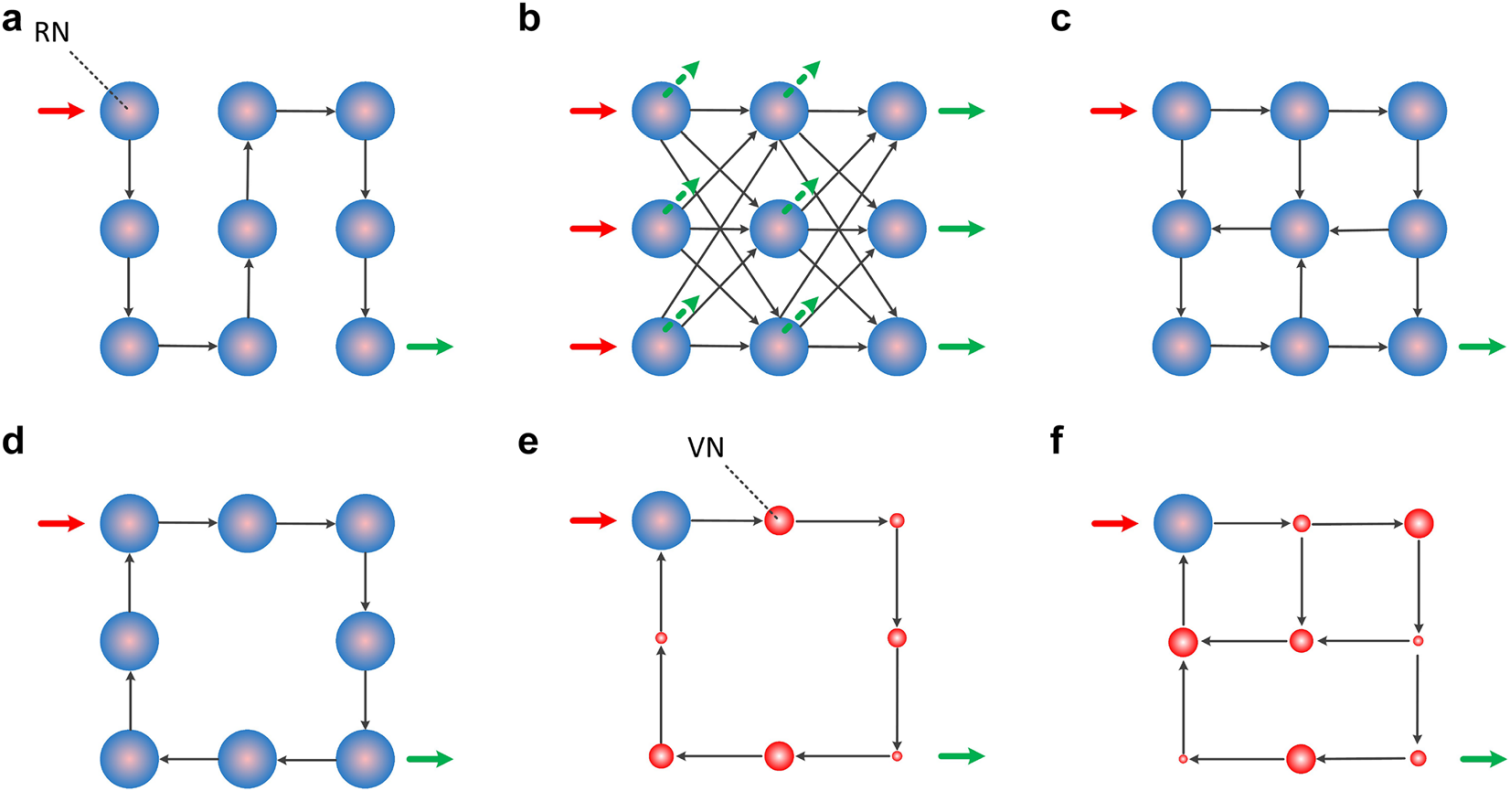
Different topologies in photonic neuromorphic computing, using physical nonlinear elements (real nodes, RN) and feedforward (a) and (b) or recurrent connectivity (c) and (d). In simplified schemes with a single physical nonlinear element and temporal delay (e) and (f), the dimensionality of the state space increases by considering weighted virtual nodes (VN). Links are unidirectional. RNs may introduce identical or different nonlinear functions. VNs are represented by different sizes to annotate their different weighing impact. Red arrows annotate the input signal. Green arrows annotate the RN or VN responses that are used for computing.

Some simple configurations that use coupled physical nonlinear elements – noted as real nodes (RN) – are shown in Figure 1, with feedforward (Figure 1a and b) or recurrent (Figure 1c and d) connectivity. These configurations are aligned with the feed-forward (FNN) and the recurrent (RNN) NN topologies which were designed for computational algorithms. In an FFN consideration, an input sequence undergoes multiple serial nonlinear transformations by the RNs and is collected at the output (Figure 1a). In this case, the dimensionality of the input information (*D*_i_) is preserved at the output and is equal to 1 (*D*_o_ = *D*_i_ = 1). When considering a layered FNN structure (Figure 1b), multiple features of the input information are introduced at different RNs of the input layer, while the nonlinear responses are obtained at the output layer of the network. In the examples of Figure 1, the dimensionality of the input information is preserved (*D*_o_ = *D*_i_ = 3). But one can use additional RNs to introduce the input information or to measure additional RN responses, in all the presented topologies. For example, in Figure 1b, if all RNs are considered to participate at the output layer (dashed and green arrows), the dimensionality of the feature space that is obtained at the output is expanded (*D*_o_ = 9 > *D*_i_ = 3). To the opposite end, one can consider only one response of the RN topology and reduce the output information dimensionality to *D*_o_ = 1 < *D*_i_ = 3. In various DSP approaches that apply to optical communications, data reduction is rather common to endorse fast computations at the post-processing stage.

The FNN connectivity topologies of Figure 1a and b, represent a system without any memory of the previous inputs. In algorithmic computing this is straightforward. However, when considering physical devices and addressing time-dependent computing tasks, things become more complicated. The relation between the input update rate and the characteristic time response of the physical device defines if the system has memory or not. If the input is updated slower than the response time of the RN, the RN reaches a steady state before the next input arrives and the system has no memory. But, if the input is updated faster than the response time of the RN, the operating condition of the RN is characterized by transient response states [82]. In this case, the system has a short-term memory of previous inputs and is not a pure FNN.

An extension of the memory properties in such topologies is implemented by recurrent connectivity. The RN responses are re-introduced as an input, after some time delay, at some RNs of the network. Such RNN configurations are shown in Figure 1c and d. The connectivity links between RNs may be introduced by following random, mesh, or structured topologies. Additionally, the input and the output layer of such networks are not strictly defined for information processing tasks. The information can be introduced at a single or multiple RNs of the network. Similarly, the number of the RN responses that can be used for the computing task may vary. The simplest scenario to follow is a single input/output transformation, where one RN acts as the input layer and one RN acts as the output layer. In terms of memory capability, the different connectivity delays define the memory length that is retained by the network. However, this dependency comes along with the nonlinear transformations that apply in each RN, which also shape the memory length and type (linear or nonlinear memory). While in the general RNN notation, the connections of all layers are trained, in the reservoir computing (RC) approach – also stated as echo-state network [42] or liquid-state machine [43] – offered a significant simplification for actual implementations. Links can be established randomly or with some predefined rules, and only the responses that contribute to the output layer are trained. This computational topology was initially evaluated in a wireless channel equalization task [83].

A significant simplification of the RC came with the proposal to reduce the RN elements and substitute them with virtual nodes (VNs) along the recurrent connectivity links (Figure 1e and f). This approach reduces the number of nonlinear physical elements and benefits the hardware implementations [82]. However, the VNs definition becomes meaningful when it differentiates from just being delayed copies of the RN response. Thus, every VN is always weighted with a fixed value that defines the connectivity link strength in the network. In time-multiplexed information processing systems, this weighing vector is applied as a pre-processing, masking layer [82]. The simplest recurrent topology comes with a single link that connects the RN with its time-delayed response (Figure 1e). The use of multiple links of different delays and weighting can extend the computing capabilities, entering into DNN topologies [84].

These different network variants were constructed with assorted photonic and optoelectronic systems, as will be presented in the next sections. Most of them have shown remarkable capabilities in solving classification and prediction tasks. However, these hardware systems provide at their output a set of responses that correspond to a specific input. Yet, the signal acquisition by itself is not sufficient to provide classification or prediction results. At the output layer, the obtained signal responses are used to train a linear classifier and calculate its weighting factors. The selection of this kind of classifier is justified by its simplicity. It requires only the multiplication of the weighting factors with the corresponding output nodes and their overall summation. Thus, its implementation also in high-speed hardware does not seem unrealistic. Nevertheless, most of the works that consider photonic and optoelectronic approaches use an offline algorithmic implementation of the linear classifier.

### 3.2 Reservoir computing with single nonlinear photonic element

**Figure 2:**
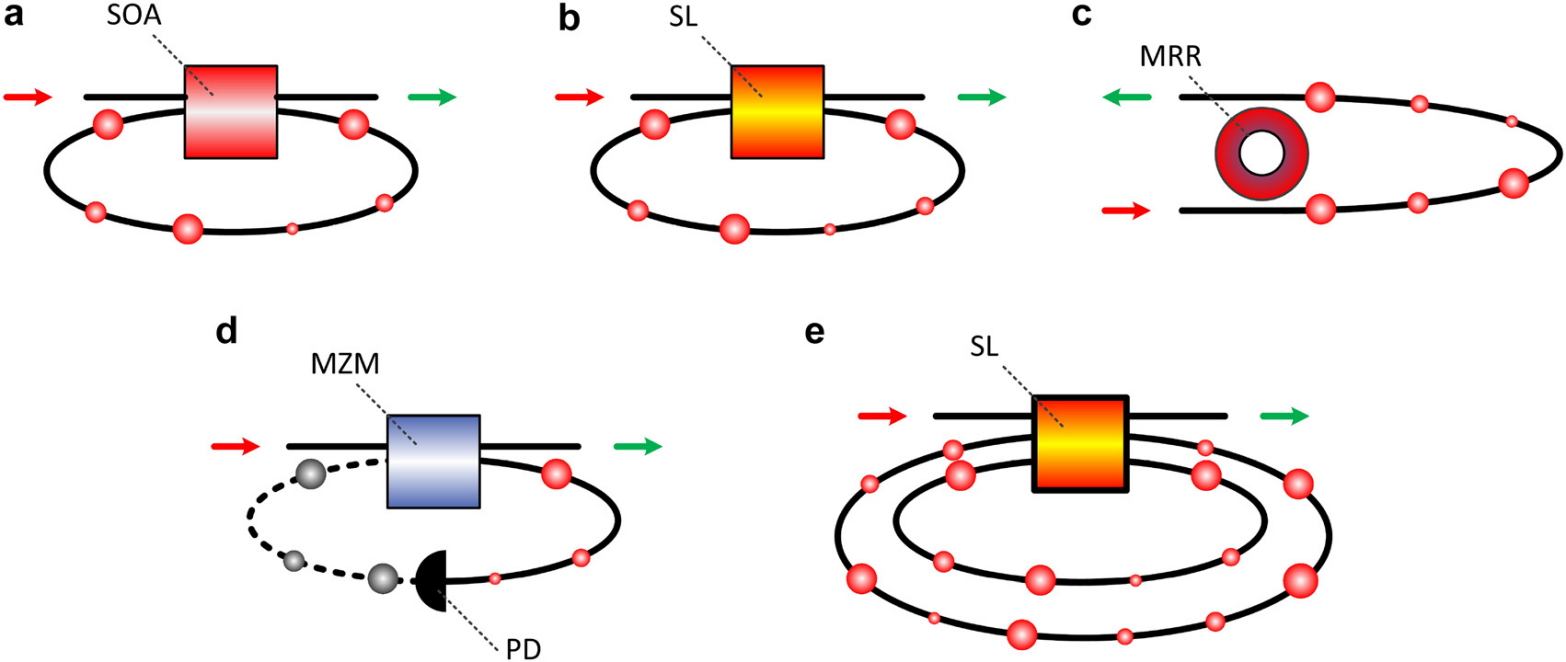
All-optical and optoelectronic topologies that are used in reservoir computing with a single real node and feedback loops, for optical information processing. All-optical systems that exploit the nonlinear operating regime of (a) semiconductor optical amplifier (SOA), (b) semiconductor laser (SL), and (c) microring resonator (MRR) with single external optical delay. Optoelectronic systems (d) that exploit the nonlinearity of Mach–Zehnder modulators (MZM) in presence of an optical and electrical delay, including optoelectronic conversion. (e) SL with multiple optical delays. PD: photodetector. Straight connection line: optical paths. Dashed connection line: electrical paths.

In the last decade, many of the proposed photonic and optoelectronic topologies included nonlinear elements that exhibit frequency responses that are well beyond the GHz regime. In this section, I review those systems that appear as promising candidates for implementing on-the-fly nonlinear transformations of analog signals that propagate in the fiber-optic communication links. There are various devices and systems that exhibit nonlinear operations when subject to feedback loops. From the experience of using ANNs in numerous applications, one may conclude that defining a precise nonlinear transfer function to describe the RNs might not be so important. This relaxes the conditions to consider different physical systems as RNs, that exhibit sigmoid, hyperbolic tangent, sinusoidal, ReLU, or other types of even more complex nonlinear functions. In parallel, it is convenient to consider physical devices and systems that have common interfaces with the associated tasks that will be used. When considering optical communication systems, it is natural for one to search the available solutions in the pool of components used in this technological field. And it is remarkable that many of the key components which are used in the optical communication systems are forced to operate in a linear regime, while they are inherently nonlinear: semiconductor lasers (SL) that operated near their optical emission threshold; semiconductor optical amplifiers (SOAs) in their full range of operation – from non-emission to gain saturation; Mach–Zehnder intensity modulators with a sinusoidal optical response; or, photonic mirroring resonators (MRR) that auto-tune their resonance frequency, depending on the circulating optical power. These devices have been part of the telecommunications industry for decades now. And all of them were considered lately for photonic signal processing, either in RC topologies with discrete optical, fiber-based, and microwave components, or in photonic integrated circuit (PIC) designs. Additionally, most of them have been also considered in the RC simplification of [82], as the single nonlinear element in all-optical (AO) or optoelectronic (OE) topologies with single or multiple feedback loops (Figure 2).

One of the first experimental realizations of AO single-RN RC with optical feedback was implemented using an SOA device (Figure 2a). It was based on a nonlinear all-optical loop operating in an incoherent regime, while the nonlinearity was provided by the saturation gain effect of the SOA [85]. A subsequent work employed the nonlinear response of an SL subject to feedback (Figure 2b) [86]. In both systems, the information was introduced into the system in the optical domain, by modulating the optical emission of a second SL. Along the delay line of the feedback loop, different numbers of VNs were defined (50 in [85] and 388 in [86]). An important difference between these two systems was the speed capability to process the information. Since the information encoding followed the principles presented in [82], the processing speed was directly associated with the time delay of the feedback loop. In [85], the delay was *τ* = 7.9437 µs, while in [86] it was almost two orders of magnitude shorter (*τ* = 77.6 ns). These two systems had also a difference in the response time of the nonlinearity. In [85], the response of the SOA was slowed down by using a low pass filter, to introduce the connectivity between the VNs, as discussed in Section 3.1. But this limitation can be lifted when considering much shorter time delays and temporal distances between the VNs. In [86], the response of the SL is at the GHz range and the temporal distance between the VNs was only 200 ps. For successful information processing, a significant number of different nonlinear transients is required. This number can be from several tens and up to many hundreds of VNs. Inspired by these topologies, many subsequent works aimed at improving the performance of classification and prediction tasks. However, the time multiplexing of a large number of VNs has an impact on the overall information processing speed of the system.

From an energy consumption viewpoint, the use of active elements for introducing nonlinear signal transformations, such as the SOAs and the SLs, is energy inefficient compared to photonic passive solutions. MRRs were used as high-quality optical filters in photonic topologies [87] demonstrating some important attributes: they do not require energy to operate, they are miniaturized devices and they are characterized by strong nonlinearity under conditions [88]. When they are used in an RC topology, in the absence [89] or presence [90] of an optical feedback loop (Figure 2c), they were very efficient in solving classification or prediction benchmark tasks.

In the last two decades, there has been a systematic and in-depth investigation of the OE oscillators, not only as a fundamental nonlinear system but also in various applications related to secure communications, microwave generation, physical random number generation, sensing, and signal processing [37], [38], [39], [40], [41]. An exceptional overview of their use in different scientific fields, including information processing, is given in [36]. These oscillators are another simple topology that conformed with the time-delay RC requirements [91–93]. One of the first demonstrations of the time-delay RC concept, with a nonlinear element and an external delay, was an optoelectronic system [91]. In the most common version of the OE oscillators, an optical signal is electrically modulated by its delayed response, via an OE device – i.e. an amplitude (MZM) or a phase (PM) modulator. The delayed response is obtained by the combination of the optical and electrical paths. After photodetection, it modulates back the optical signal (Figure 2d). Attributes such as the high gain of the photoreceiver, the modulator nonlinearity, and the time delay of the conversion path, allow high-dimensional dynamical behaviors to be observed [94]. Simply by selecting high-bandwidth components to build this system, one can achieve a broadband operation up to tens of GHz. Thus, these oscillators can be responsive to ultrafast signals and perform nonlinear processing at equivalent speeds.

In the simplest AO and EO RC topologies, only a single external feedback loop was considered. Already, this resulted in systems with complex behavior. However, some dynamical properties may be further enriched by considering multiple delays (Figure 2e). In [95], the maximization of the linear memory of an RC topology, based on an Ikeda oscillator, was achieved through multiple delays with unequal length. In [96], the use of double feedback OE loops led to improved performance in various classification and prediction tasks. Topologies based on a single nonlinear element with multiple static [97–99] or modulated [84] loops also showed improved performance in classification tasks.

The different photonic systems that appear in Figure 2, were initially demonstrated with discrete components. Soon, several designs were transferred to photonic integrated platforms. Photonic integrated circuits (PICs) with simple and robust designs were fabricated in the past, exhibiting complex dynamical behaviors. For example, in [100] a PIC that consisted of a distributed-feedback semiconductor laser, an optical amplifier, a phase modulator, and a short external cavity for optical feedback, was shown to exhibit rich dynamical states of operation. Similar PIC versions have been fabricated experimentally [101] for applications in secure optical communication [102], random number generation [103, 104], and secure key distribution [105]. In [106], this structure was used in an RC architecture, for time-series prediction and nonlinear channel equalization tasks. An indium-phosphide PIC that combined active and passive elements – a semiconductor laser with an external cavity of 5.4 cm and an SOA – was demonstrated in [107]. In its feedback delay time of 1170 ps, 23 VNs were accommodated, achieving a processing speed of 0.87 GSa/s.

### 3.3 Reservoir computing with multiple linear or nonlinear photonic elements

**Figure 3:**
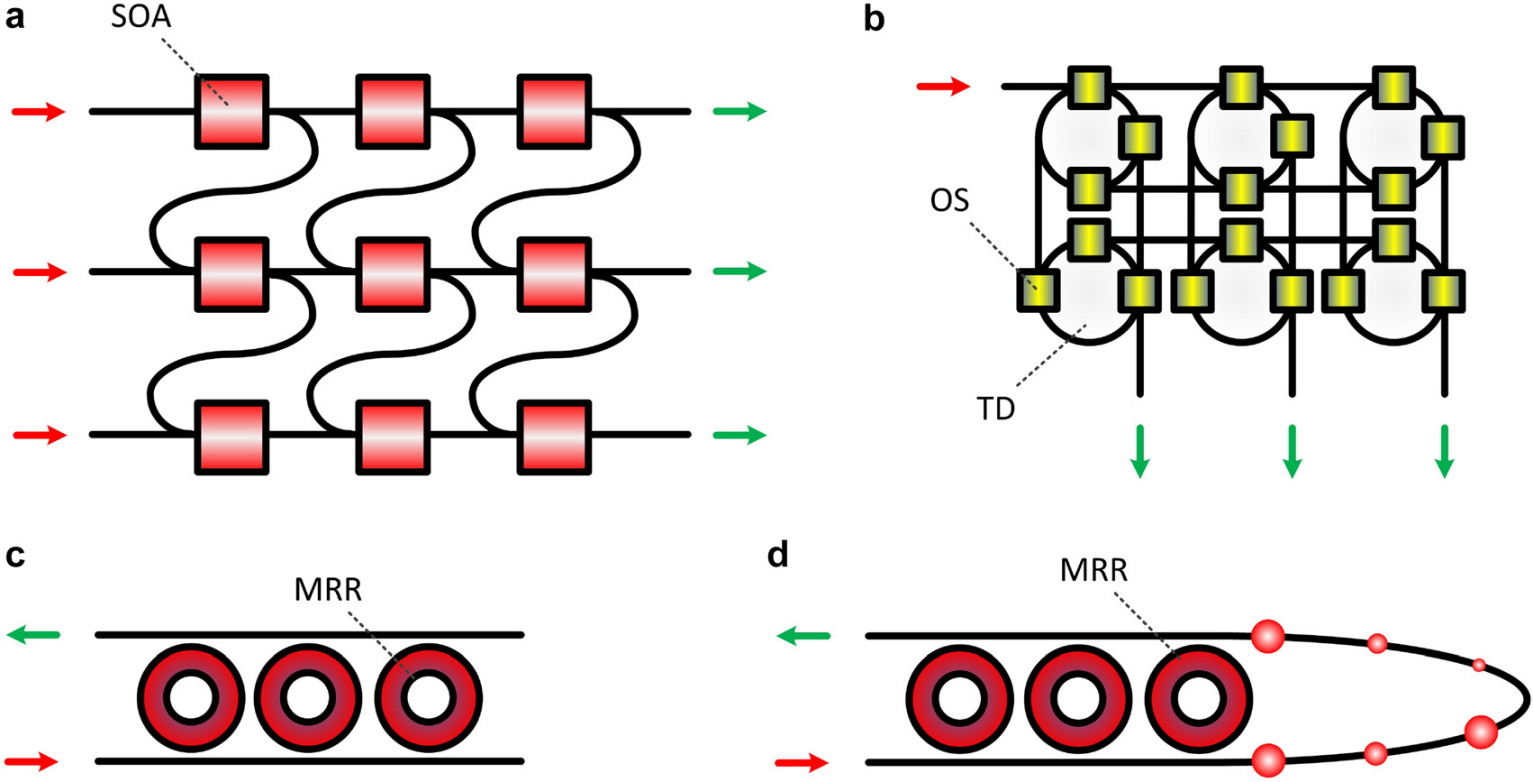
All-optical nonlinear (a), (c) and (d) and linear (b) topologies, with multiple real nodes, for optical information processing. (a) Mesh network of multiple nonlinear SOA. (b) Mesh network of multiple optical splitters (OS) and different time delays (TD). (c) MRR array with different MRR elements and intra-MRR delays. (d) MRR array with different MRR elements and external optical delay.

The simplest topologies that use an optical nonlinearity and external time-delayed feedback were not the first ones proposed to build a photonic RC. The first proposal was in 2008 [108], where a network of coupled SOAs was considered as the basic building block for the reservoir. The proposal included SOA connections that led to either an FFN or an RNN topology. A similar scheme, based on a swirl topology, was investigated numerically in detail [109]. The general concept of a coupled SOA network in an FNN topology is shown in Figure 3a. The number of RNs that participate at the output layer may differ. In this example, the input and output layers consist of 3 RNs. In [108], a different approach was used, with a single RN forming the input layer and all the RNs participating in the output layer. In [110], a swirl topology of silicon-on-insulator (SOI) MRRs was numerically investigated and used to evaluate a classical nonlinear Boolean task (delayed XOR). In [111], a design with multiple semiconductor lasers arranged in parallel on a PIC RC was proposed and numerically investigated, solving tasks like chaotic time-series prediction task, memory capacity, and nonlinear channel equalization. In [112], a scalable on-chip photonic RC design with coherent linear delay lines was proposed. This design targeted a scalable and ultrafast computing structure, exploiting an ultra-wide optical bandwidth via wavelength division multiplexing. All these works employed photonic integration, since combining multiple standalone devices is much more complicated than stacking them in integrated designs.

The first photonic RC implementation with multiple RNs included just waveguide time delays in a silicon-on-insulator (SOI) platform, in a passive and energy-efficient design [113]. This linear device was combined with the nonlinearity at the photodetection stage to solve arbitrary Boolean logic operations. In a schematic generalization of this concept (Figure 3b), multiple optical splitters define different delays among different routes of the RN network. Again here, the RNs that are assigned to the input and the output layer may differ.

Configurations such as the ones in Figure 3a and b face significant scalability limitations in photonic integration designs. The number of RNs that can be included in a design may increase substantially, only when considering devices with small footprint. But most importantly, the assignment of many RNs to the output layer results in a detection bottleneck; the response of each RN that participates at the output layer requires a photodetection stage to be measured. In [113], the responses of only 11 RNs were measured to train and test the linear classifier. In most of the RC configurations that were studied numerically, many tens or even hundreds of RNs were used at the output layer to feed a classifier. These numbers appear very challenging to be transferred in real implementations. Nevertheless, the possibility to process the output layer signals at the optical domain and implement a linear classifier directly on-chip is not unmanageable. Designs that were proposed in a different context of optical signal weighting and summation [114–117] can apply also here, with only slight adaptations. In those designs that consider linear coupling of optical signals, the photodetection at the output of the classifier is used to introduce the nonlinearity. Finally, schemes that combine both multiple RNs and time multiplexing in a single input – single output (SISO) RC may offer improved computational performance, compared to a single RN topology. An example of such designs can incorporate MMRs in a Scissor topology [118], in the absence or presence of external optical delays (Figure 3c and d).

### 3.4 Photonic neuromorphic systems for channel equalization

The nonlinear equalization of a signal that propagates in a transmission path has been a problem for decades in the signal processing community [119, 120]. A rather specific task, that was studied first in [121] for wireless transmission, resulted as a popular benchmark task for the echo-state network community [83]. The propagation channel is modeled by a linear system with a memory length equal to 10, followed by a memoryless nonlinearity that is subject to noise. The input to the channel is an independent and identically distributed random sequence *d*(*n*) with values: {−3, −1, 1, 3}. The input signal goes through the linear channel first, providing an output signal:

(1) $$\begin{array}{l} q\left(n\right)=0.08d\left(n+2\right)-0.12d\left(n+1\right)+d\left(n\right)+0.018d\left(n-1\right)-0.1d\left(n-2\right)+0.091d\left(n-3\right)-0.05d\left(n-4\right)+0.04d\left(n-5\right)+0.03d\left(n-6\right)+0.01d\left(n-7\right) \end{array}$$

At the next stage, this signal undergoes a nonlinearity with where *v*(*n*) is an independent and identically distributed Gaussian noise with zero mean, which is adjusted in power to yield an SNR between 16 and 32 dB:

(2) $$u\left(n\right)=q\left(n\right)+0.036q{\left(n\right)}^{2}-0.011q{\left(n\right)}^{3}+v\left(n\right)$$

The computing task is to reconstruct the input *d*(*n*) from the output *u*(*n*) of the nonlinear channel. The task performance is usually evaluated by the symbol error rate (SER). This task has been used extensively to demonstrate the performance capabilities of the various photonic RC topologies. Here, I summarize the configurations that were used to solve this benchmark task. An overview of their attributes and performances is given in Table 1.

**Table 1:** Photonic neuromorphic systems based on RC and applied on the nonlinear channel equalization benchmark task. AO: all-optical system. OE: optoelectronic system. PIC: photonic integrated circuit. SRL: semiconductor ring laser. Exp.: experimental investigation. Num: numerical investigation. T: time delay. L: length.

System type	Parameters	Dimensionality	Performance	References
OE (Exp.)	MZM and PD nonlinearity, desynchronized input with time delay, *T* = 8.504 ms	50	SER = 1.3 × 10^−4^ for SNR = 32 dB	Paquot et al. [91]
AO (Exp.)	SESAM nonlinearity, desynchronized input with time delay, *T* = 8.0073 μs	50	SER < 10^−4^ for SNR = 32 dB	Dejonckheere et al. [122]
OE (Num., Exp.)	Coherent passive cavity, desynchronized input with time delay, *T* = 1.13209 μs	50	SER < 2 × 10^−5^ for SNR = 32 dB	Vinckier et al. [123]
OE (Num., Exp.)	MZM and PD nonlinearity, desynchronized input with time delay, *T* = 247.2 μs	Variable	SER < 3 × 10^−3^ for SNR = 28 dB	Ortin et al. [124]
AO (Num.)	SRL nonlinearity, parallel task processing, *T *= 4 ns	200	SER = 2.3 × 10^−3^	Nguimdo et al. [125]
OE (Exp.)	MZM and PD nonlinearity, desynchronized input with time delay, analog input and output layer, *T* ≈ 8.4 μs	47	SER = 10^−4^ for SNR = 32 dB	Duport et al. [126]
OE (Exp.)	MZM and PD nonlinearity, desynchronized input with time delay, FPGA training, *T* = 7.94 μs	50	SER = 5.71 × 10^−6^ for SNR = 32 dB	Antonik et al. [127]
AO (Num.)	VCSEL nonlinearity with optical feedback and injection, *T* = 0.64 ns	32	SER = 3 × 10^−5^ for SNR = 32 dB	Vatin et al. [128]
AO (Exp.)	VCSEL nonlinearity with optical feedback and injection, low system SNR = 12 dB, *T* = 39 ns	390	SER = 1.5 × 10^−2^ for SNR = 12 dB	Vatin et al. [129]
AO (Exp.)	PIC with SL, phase section, SOA, waveguide and reflector, *T* = 256 ps	6	SER = 1.2 × 10^−2^	Takano et al. [106]
OE (Num.)	MZM and PD nonlinearity, double optoelectronic feedback	50	SER = 7 × 10^−4^ for SNR = 32 dB	Chen et al. [96]
AO (Num.)	Multiple reservoirs with SL and optical feedback, *T* = 240 ps, 38 subsystems	24	SER = 9 × 10^−4^	Sugano et al. [111]
AO (Num.)	Mutually coupled SLs with optical delay, *T* = 4 ns	200	SER = 6.7 × 10^−4^	Hou et al. [130]
OE (Num.)	Mutually coupled SLs with optoelectronic delay, *T* = 10.1 ns	100	SER = 3.3 × 10^−4^	Liang et al. [131]
AO (Num.)	SL with optical feedback, electronic injection at the input layer, T: tunable	50	SER = 4 × 10^−5^ for SNR = 32 dB	Yue et al. [132]
MMF (Exp.)	Speckle pattern, phase-driven nonlinearity, *L* = 10 m, ∼250 modes	100	SER = 2.2 × 10^−2^	Sunada et al. [133]

In [91], Paquot et al. built an experimental OE setup, with a single nonlinear RN and a delay line. The nonlinearity was introduced by an MZM in the optical path and by a PD at the OE conversion of the feedback loop. In their configuration, they considered 50 VNs, while the input encoding rate and the period of the delay line were unmatched. This shows the flexibility of transferring the RC concept into hardware implementations with feedback loops. Their system reached an SER = 1.3 × 10^−4^, for SNR = 32 dB. In [122], Dejonckheere et al. reported an all-optical implementation with a passive nonlinear element, namely a semiconductor saturable absorber mirror (SESAM). This energy-efficient design exploits a nonlinearity that was activated for low optical input power. This system reached an SER < 10^−4^, for SNR = 32 dB. In [123], Vinckier et al. presented an experimental implementation of a coherently driven passive fiber with low losses. The absence of active elements in the cavity reduced the noise sources and decreased the energy consumption of the reservoir. With this system, an SER < 2 × 10^−5^ was achieved, for SNR = 32 dB. In [124], Ortín et al. used the optoelectronic topology to investigate the impact of the recurrence of the feedback delay, compared to an ELM. An SER < 3 × 10^−3^ was achieved for SNR = 28 dB with the RC topology, showing a slightly better performance compared to the ELM. In [125], Nguimdo et al. used a semiconductor ring laser (SRL) to solve simultaneously two tasks with uncorrelated input streams. Each directional mode of the SRL was assigned to process the different tasks and one of them was a nonlinear channel equalization. The lower SER obtained was SER = 2.3 × 10^−3^. In this work, the optical feedback time delay was significantly reduced to only 4 ns. In [126], Duport et al. combined the OE reservoir with an analog input and output layer. The output of the photonic RC system was an analog electrical signal, proportional to the output requested by the task. In [127], Antonik et al. trained an OE RC system by using a simple gradient descent algorithm, programmed on a field-programmable gate array (FPGA) chip. Vatin et al. investigated numerically [128] and experimentally [129] a VCSEL device as the nonlinear element, in an all-optical scheme. In the latter, however, the optical system suffered from low SNR. This shows how the SNR requirements of a signal processing task have to align with the SNR specifications of the photonic computing system. In [106], Takano et al. used a PIC as the AO cavity, in a time delay RC approach. The limited length of the time delay (256 ps) and the 40 ps VN temporal separation, allowed only a small dimensionality expansion of the input signal, limiting the final performance. In [96], Chen et al. claimed a better RC performance by considering two optoelectronic feedback loops, with different time delays. Sugano et al. [111] expanded the concept of Figure 2b, by using multiple all-optical reservoir subsystems in a parallel design, where each subsystem received a common optical injection. Their results showed that this scheme outperforms the single laser topology with multiple delay times. In [130], Hou et al. evaluated an RC system with two mutually coupled SL, where each SL plays the role of an RN. In [131], Liang et al. evaluated the same concept but using OE conversion within the coupling loop. In [132], Yue et al. investigated the simple topology of an SL with optical feedback (Figure 2b), but with the difference of injecting the information as a biasing current modulation of the SL. Finally, in [133], Sunada et al. proposed a scheme based on a multimode fiber speckle pattern. This approach combined the use of space, wavelength, and time multiplexing to achieve high-speed, scalable, parallel processing. The nonlinearity was introduced by using fast optical phase modulation that maps phase-encoded information into speckle patterns. However, such systems are expected to be prone to phase and polarization instabilities, and obtaining a low SER is a challenge.

### 3.5 Photonic neuromorphic systems for optical header recognition

As the optical network infrastructure becomes increasingly complex in its deployment, the necessity to make reliable routing decisions is a key property. Additionally, routing decisions have to be made fast, following the data flow rate. There have been many considerations in the past employing optical techniques to aid electronic solutions in making rapid routing decisions. The 32-bit (IPv4) and 128-bit (IPv6) destination packets, that are at the forefront of a data stream, have a much higher dimensionality compared to the physical routing hardware. Thus, the decision to determine which is the outgoing port of a packet, by evaluating a smaller subset of the destination address information, is a hard task. Already from the 90s, optical systems were designed to address this challenge [134–136]. During the last decade, several photonic implementations – including RC topologies – have proven efficient for multi-bit header recognition. This task is linear and is easier to solve, compared for example to a delayed XOR task. However, when increasing the length of the header, this linear task can be also hard to solve with a simple computing scheme and a small dimensionality expansion of the input information. Nevertheless, to validate such systems as efficient optical header classifiers, the evaluation criterion is not only the classification performance. In operational network deployments, these systems have to conform with the data rates of the incoming information. In this section, I review these configurations. An overview of their attributes and performances is given in Table 2.

**Table 2:** Photonic neuromorphic systems based on RC applied on the optical header recognition task. PCC: photonic crystal cavity.

System type	Parameters	Dimensionality	Performance	References
AO (Num., Exp.)	Passive linear optical cavity, 8-bit and 5-bit header, interconnection RN delays *T*_i_ = 280 ps	36, 16	ER = 0	Vandoorne et al. [113]
OE (Num., Exp.)	3-bit header, MZM and PD nonlinearity, *T* = 2.5 ns/*T* = 9.67ns	400	WER = 1.25%	Qin et al. [137]
PCC (Num.)	6-bit header, 30 × 60 μm cavity, 1 input and 6 output waveguides	Not defined	ER < 10^−3^	Laporte et al. [138]
OE (Num.)	Mutually-coupled double OE system, 3-bit header, 8 types of 16-bit header, 4 types of 32-bit header, *T* = 2.5 ns	400	WER = 0	Zhao et al. [139]
OE (Num.)	Mutually-coupled multiple OE system, 3-bit header, 8 types of 16-bit header, 4 types of 32-bit header, *T* = 2.5 ns	400	WER = 0	Bao et al. [140]
AO (Num.)	PIC with Y junctions, multimode operation, 3-bit header	16	ER < 2 × 10^−3^	Katumba et al. [141]
AO (Num.)	4-Port swirl passive linear optical cavity, 1 type of 4-bit header	16	BER < 10^−4^	Ma et al. [142]

The first deployment of a photonic RC structure for header-type pattern recognition was reported by Vandoorne et al. in [113]. The considered integrated passive silicon reservoir with 16 RNs was evaluated in a 5-bit header classification task. In an extension with numerical investigations, they classified an 8-bit header, by considering a larger scale reservoir with 36 RNs. In [137], Qin et al. studied numerically and experimentally an OE RC topology in an identification task of optical packet headers with lengths from 3 to 32 bits. In their experimental evaluation, they achieved a word error rate of 1.25% for 3-bit headers. From a dynamical point of view, they identified the optimal operating reservoir operation, at the critical state between multiple-period oscillations and the chaos, where the maximal Lyapunov exponent becomes slightly positive. In [138], Laporte et al. proposed the use of a photonic crystal cavity (PCC) as the photonic reservoir, with one waveguide acting as the input port and six more waveguides as the collecting information ports. With this scheme, they reported ER < 10^−3^, for 6-bit headers and bit rates up to 100 Gb/s. While this passive design allows ultrafast optical header processing, an experimental demonstration is a big challenge. In [139], Zhao et al., studied a design of mutually-coupled dual optoelectronic cavities, with MZM and PDs as the nonlinear elements. They showed up to a 32-bit header classification with no errors. However, in their evaluations for headers above 3 bits, they considered only a small subset of the possible patterns. In an extension of this work, Bao et al. [140] considered an increased number of mutually coupled dual OE cavities, with multiple input-output RC interface points, and investigated the impact of SNR on the classification performance. In [141], Katumba et al. studied numerically a PIC structure with multimode Y junctions as a reservoir, in a 16-RN topology with an all-input – all-output operation. Their system provided ER < 2 × 10^−3^ for a 3-bit header recognition task while considering data input rates of 32 Gb/s. Finally, in [142], Ma et al. considered the passive linear cavity of [113], in a swirl topology with 4 ports to identify one type of a 4-bit header. This investigation mainly focused on the impact of the quantization resolution and noise to the final performance. When it comes to the computation and the application of the weighting factors to an optical system, the available SNR may not be as high as in the electronic domain. This work showed that optical topologies that suffer from significant noise and have a very low weighing resolution, can still deliver performance very close to full-resolution weighting elements.

### 3.6 Photonic neuromorphic systems for data recovery

**Figure 4:**
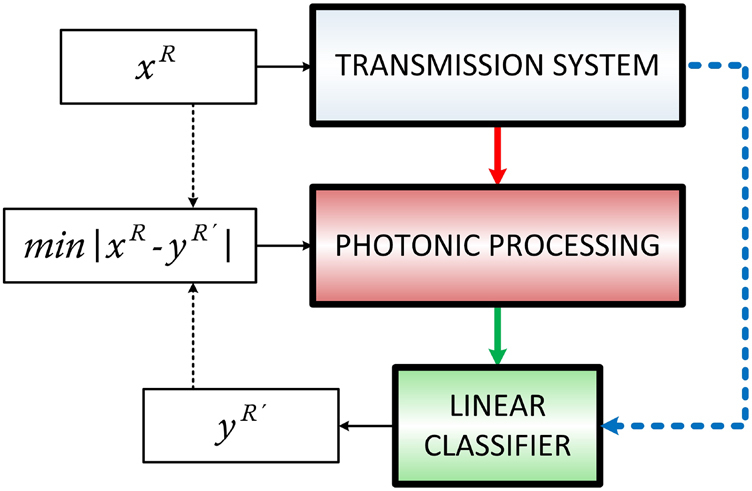
Block diagram of data recovery with photonic processing after optical transmission. The data stream *x* is encoded with data rate *R* and sent for optical transmission. The received signal after transmission is introduced at the photonic processing system (red arrow). The obtained signal response is used to train a linear classifier (green arrow) that predicts the data stream *y*. In case that the photonic processing introduces speed penalty, the predicted data stream is obtained with a slower data rate *R*´. The minimization of the error |*x*–*y*| is obtained by parameter optimization of the photonic processing. The final performance is commonly benchmarked to the performance of the same classifier trained by the received signal from transmission (blue line).

In Section 2, different DSP approaches that apply in optical communication systems to mitigate linear and nonlinear effects were presented. However, some of the key functionalities that contribute to this mitigation can be also obtained by photonic systems. The memory properties of the previous states of a signal can be introduced by optical or optoelectronic recurrences. They can be also introduced in the system via the transient responses of a bandwidth-limited operation. In parallel, nonlinear transformations of the input signal’s states can be obtained by various AO and OE devices and subsystems, as presented in Sections 3.2 and 3.3, under the RC framework. Some of these topologies were considered in the last few years, for linear and nonlinear fiber transmission channel equalization, and follow the block diagram logic of Figure 4. An overview of their attributes and performances is given in Table 3.

**Table 3:** Photonic neuromorphic systems based on RC applied on the signal recovery. *T*_n_: time delay in numerical simulation. *T*_e_: time delay in experimental implementation. *T*_AO_: time delay in AO system. NOLM: nonlinear optical loop mirror.

System type	Parameters	Dimensionality	Performance	References
AO (Num., Exp.)	SL with optical feedback, OOK encoding, 25 Gb/s, 50 km, *T*_e_ = 66 ns	66	BER = 1.8 × 10^−4^	Argyris et al. [143]
AO (Exp.)	SL with optical feedback, OOK encoding, 10 Gb/s, 4000 km, *T*_e_ = 66 ns	66	BER = 1.7 × 10^−3^	Argyris et al. [143]
AO (Num., Exp.)	PAM4 encoding, 28GBaud, 27 km/21 km, *T*_n_ = 0.8 ns/1.6 ns, *T*_e_ = 66 ns	32	BER = 2 × 10^−3^	Argyris et al. [144]
AO (Num., Exp.)	PAM4 encoding, 56GBaud, 5.5 km/4.6 km, *T*_n_ = 0.8 ns/1.6 ns, *T*_e_ = 66 ns	32	BER = 2 × 10^−3^	Argyris et al. [144]
AO, OE (Num.)	Comparison SL with optical feedback vs. MZM and PD nonlinearity, OOK encoding, 25 Gb/s, 50 km, *T*_AO_ = 10 ns	400	BER < 10^−3^	Argyris et al. [145]
AO (Num.)	SL with optical feedback, QPSK encoding, 28GBaud, 180 km, *T* = 0.96 ns	80	BER ∼ 10^−3^	Estébanez et al. [146]
AO-MRR (Exp.)	SOI-MRR, PAM4 SSB encoding, 56GBaud, 100 km	16	BER = 2 × 10^−4^	Li et al. [147]
AO (Exp.)	SL, PAM4 SSB encoding, 56GBaud, 100 km	24	BER = 3 × 10^−4^	Estébanez et al. [148]
AO (Num.)	FP laser with optical injection and feedback, multiple modes, PAM4 encoding, 25GBaud, 50 km, *T* = 0.24–1 ns	12–50	BER < 10^−3^	Bogris et al. [149]
AO (Exp.)	Si-PIC with MMI splitters, OOK encoding, 32 Gb/s, 25 km	32	BER = 10^−5^	Sackesyn et al. [150]
NOLM (Num.)	QAM16-256 encoding, 30GBaud, 100 km	32	∼2 dB gain vs. linear equalizer	Sorokina et al. [151]
NOLM (Num.)	QAM64-256 encoding, 30GBaud, 100 km, 10 dB launched optical power/QAM256 encoding, 30GBaud, 1 km, 20 dB launched optical power/QAM256 encoding, 30GBaud, 0.5 km, 20 dB launched optical power	32	∼2–3 dB gain vs. linear equalizer/BER = 10^−3^/BER = 10^−3^	Sorokina et al. [156]
NOLM (Num.)	5-Channel QAM64 encoding, 32GBaud, 10 × 80 km	16	1.2 dB gain vs. linear equalizer	Sorokina et al. [157]

In [143], Argyris et al. used an AO reservoir with an SL and external optical feedback, to demonstrate experimentally data recovery from signals that have undergone a simulated fiber optic transmission at 1550 nm. Specifically, they used two-level encoding (on-off keying, OOK) in amplitude, for two types of transmission systems. The first corresponded to a short-reach transmission system with a 25 Gb/s encoding rate and 50 km of standard single-mode fiber (SSMF). The second corresponded to a long-haul transmission system with a 10 Gb/s encoding rate of 4000 km, with optical amplification and chromatic dispersion stages every 100 km, for power loss and chromatic dispersion compensation. The photonic reservoir had 66 VNs, while the performance of the classifier was optimized by considering also the reservoir’s responses of neighboring bits. The BER improvement for both systems was more than one order of magnitude, compared to the performance of a linear classifier that was applied to the same signal from transmission. This work also showed numerically that the RC dimensionality can be reduced significantly, if a high sampling is applied at the input signal. The same photonic RC system was numerically evaluated from the previous group, for data recovery of 4-level, pulse amplitude modulation encoding (PAM4), at 28GBaud rate and 27 km, and at 56GBaud rate and 5.5 km [144]. These transmission lengths allowed to obtain a BER below the hard-decision forward error correction threshold. However, in an experimental evaluation, the transmission lengths of these two systems were reduced to 21 and 4.6 km respectively, to replicate the same performance. In [145], Argyris et al. made a numerical comparison between two photonic reservoir computing systems for the same fiber transmission data recovery task. The OE system offered slightly lower BER values, compared to the AO system with SL and optical injection. In [146], Estébanez et al. expanded the bandwidth operation of the SL with optical feedback in an RC architecture, to exploit transient reservoir responses as fast as 12 ps. This RC topology was used for data recovery in a 180 km coherent transmission system with 28GBaud quadrature phase-shift keying (QPSK) encoding. The obtained results showed the importance of the fast time response of the photonic nonlinear system when processing very fast input signals. All these photonic systems processed signals that were obtained from numerically simulated transmission systems. In [147], Li et al. utilized the signals from an experimental dense wavelength division multiplexed (DWDM) 100 km-long fiber transmission system, with PAM4 single-sideband (SSB) encoding at 56GBaud, to train a photonic RC implementation based on an SOI MRR. In this case, the nonlinearity was introduced by the distributed 3rd-order, free-carrier, and thermal nonlinearities along the MRR. The BER obtained was 2 × 10^−4^, for a transmission system with OSNR ∼ 36 dB. The same experimental transmission system was also evaluated by Estébanez et al. [148] by using an AO RC implementation that included an SL nonlinearity, with similar BER performance. Both photonic RC topologies showed, however, that they cannot offer efficient equalization, in presence of strong nonlinear effects. Some possible reasons for that are the limited dimensionality expansion of the input signal and the limited inherent fading memory of the reservoir. Bogris et al. [149] considered Fabry–Perot (FP) lasers and exploited their longitudinal modes for parallel processing. By exploiting both injection locking and optical feedback, they investigated numerically an RC scheme for data recovery from a 50 km transmission system with 25Gbaud PAM-4 encoding. In [150], Sackesyn et al. presented an experimental implementation of a Si-PIC reservoir with a swirl architecture of 32 RNs, in a 4×8 configuration. Each RN was a 3×3 multimode interferometer (MMI) splitter. They used this device to mitigate the nonlinear distortions of a 32 Gb/s OOK encoded signal, in a 25 km fiber transmission system. A BER as low as 10^−5^ was obtained, even at very high launched optical power (18 dBm). These photonic RC topologies used localized nonlinearity from an AO or OE nonlinear system. Sorokina et al. in [151] used the distributed nonlinearity from a fiber nonlinear optical loop mirror (NOLM) in an ESN approach [152] to mitigate linear and nonlinear effects in fiber transmission. This technique, also known as nonlinear Sagnac interferometry, has been proposed in the past for optical switching [153], signal demultiplexing [154], and all-optical gates [155]. Sorokina et al. exploited both high bandwidth and dual-quadrature signal processing to mitigate signal distortions of a 100 km single-span transmission with 30GBaud QAM encoding with up to 256 levels, in a numerical investigation. In [156], Sorokina et al. expanded this investigation to higher-order QAM formats – up to 1024 levels – and they improved the performance of the QAM256 encoding to ∼3 dB gain. Finally, in [157] they considered a fiber link of 800 km with intermediate amplification stages, with a 32GBaud QAM64 encoding and 5 WDM channels, reporting a 1.2 dB gain compared to a linear equalizer.

Many of these photonic neuromorphic architectures present appealing properties to address signal processing tasks in data recovery for optical transmission systems. However, there are still significant challenges to address, before placing them as comparable solutions to algorithmic DSP. The RC fundamental concept is based on the dimensionality expansion of a complex signal so that the final classification becomes more accurate. However, this goes in the opposite direction from data reduction, which is what most of the DSP algorithms aim at. For the RC concept to be functional in the framework of real-time data recovery in optical communications, the following requirements need to be fulfilled: (a) the input layer of the photonic RC can follow the sampling rate of the encoding used at the transmission system; (b) the photonic system has sufficient bandwidth to respond – in terms of providing an efficient nonlinear transformation – to such fast encoding; (c) the processing time per unit of information is equal to the encoded information duration; and (d) the output layer classifier performs on-the-fly weight multiplication and summation. All the demonstrations summarized in Table 3 have been implemented offline, since at least one of the above requirements was not met. Nevertheless, there is no fundamental reason that an appropriately designed photonic system, that conforms with all the above requests, cannot be a real-time processing unit for data recovery tasks.

### 3.7 Photonic neuromorphic systems for modulation format identification

Besides the DSP solutions presented in Section 2 for modulation format identification, there were also a few proposals last year, based on the computing concepts of Figures 2 and 3, to discriminate and identify several optical modulation formats in real-time [158, 159]. Here, I review the works that address this topic with photonic RC topologies. An overview of their attributes and performances is given in Table 4.

**Table 4:** Photonic neuromorphic systems based on RC applied on the modulation format identification.

System type	Parameters	Dimensionality	Performance	References
AO (Num.)	3 modulation formats	300/400	Accuracy: 95%	Cai et al. [160]
OE (Num.)	11 modulation formats	400	Accuracy: 89.85%	Dai et al. [161]

Cai et al. in [160] presented an all-optical RC system, based on the SL with optical feedback topology, for modulation format identification. The system processed the representative features from the asynchronous amplitude histograms of three types of modulation signals (OOK, differential phase-shift keying, and QAM). They reported an identification accuracy of >95% after optimization of the photonic RC system. Dai et al. in [161] used an optoelectronic RC system to classify 11 analog and digital formats of IQ modulating signals, obtained from the DeepSig RadioML dataset. They showed a classification accuracy of 89.85%, which is better than the state-of-the-art while adopting a simpler architecture.

### 3.8 Discussion

The neuromorphic designs that exploit an unprecedented level of parallelism are already demonstrated in electrical circuits (Intel’s Loihi, IBM‘s TrueNorth, etc.). These prototypes show a clear pathway for the near future. Appropriate designs will be transferred – hopefully soon – to photonic platforms with the appropriate interfaces for ultrafast and energy-efficient signal processing []. Most of the neuromorphic photonic systems that are presented in the literature do not fulfill yet the requirements for real-time signal processing, which is critical for data recovery. But besides that, there are several other critical challenges to be addressed, such as the energy consumption, the computational precision, and the design scalability for computations of increased complexity. The existing data centers that coordinate the global data flow from optical communication systems require massive amounts of electrical energy. A new generation of transceivers is required that will drastically reduce the energy footprint of the signal processing stages and will minimize the cooling requirements. To this end, the photonic integrated topologies that consider only passive photonic elements, such as optical splitters, interferometers, and microring resonators, along with all-optical designs, turn to be more energy-efficient. However, they suffer from scalability limitations. The optical losses introduced by such photonic elements attenuate the optical signal rapidly. Thus a compromise between energy efficiency and scalability may be considered, by combining passive and active components. In terms of computational precision, this is directly related to the extinction ratio of the obtained analog signals at the output and the dynamic operating range of the photonic systems. As analog systems are more susceptible to noise, it is a real challenge to improve the current capabilities. Still, the computational precision will never meet the one obtained by digital systems. But it may converge to an acceptable level, which will allow all other advantages of neuromorphic photonics to prevail.

## 4 Conclusions

In this work, I reviewed various neuromorphic photonic approaches that respond to different challenges of modern optical communication systems. Simple algorithms of low complexity, implemented in the digital domain, are currently the established methods to address these challenges. More advanced algorithms, that endorse machine learning and neural networks have shown their potential, but they are too complex and computationally demanding to support the modern data encoding rates. These architectures, however, will be dominant soon in the monitoring and management of optical networks. In this increasingly demanding environment, novel solutions are needed to deal with problems of further increased complexity. Neuromorphic photonics and especially hardware reservoir computing have given numerous examples of addressing challenges that relate to optical header recognition, data recovery from transmission systems, and modulation format identification. Although an established and holistic technology for real-time solutions has not been presented so far, to compete with the low-complexity digital signal processing tools, the recent advances aim in this direction.
